# Dynamic Visual Acuity, Vestibulo-Ocular Reflex, and Visual Field in National Football League (NFL) Officiating: Physiology and Visualization Engineering for 3D Virtual On-Field Training

**DOI:** 10.3390/vision8020035

**Published:** 2024-05-17

**Authors:** Joshua Ong, Nicole V. Carrabba, Ethan Waisberg, Nasif Zaman, Hamza Memon, Nicholas Panzo, Virginia A. Lee, Prithul Sarker, Ashtyn Z. Vogt, Noor Laylani, Alireza Tavakkoli, Andrew G. Lee

**Affiliations:** 1 Department of Ophthalmology and Visual Sciences, University of Michigan Kellogg Eye Center, Ann Arbor, MI 48105, USA; 2 McGovern Medical School, UT Health Houston, Houston, TX 77030, USA; 3 Department of Ophthalmology, University of Cambridge, Cambridge CB2 1TN, UK; 4 Moorfields Eye Hospital, NHS Foundation Trust, London EC1V 2PD, UK; 5 Human-Machine Perception Laboratory, Department of Computer Science and Engineering, University of Nevada, Reno, NV 89557, USA; 6 Texas A&M School of Medicine, Bryan, TX 77807, USA; 7 Department of Biology, University of Virginia, Charlottesville, VA 22903, USA; 8 Dean McGee Eye Institute, University of Oklahoma College of Medicine, Oklahoma City, OK 73104, USA; 9 Department of Ophthalmology, Blanton Eye Institute, Houston Methodist Hospital, Houston, TX 77030, USA; 10 The Houston Methodist Research Institute, Houston Methodist Hospital, Houston, TX 77030, USA; 11 Departments of Ophthalmology, Neurology, and Neurosurgery, Weill Cornell Medicine, New York, NY 10065, USA; 12 Department of Ophthalmology, University of Texas Medical Branch, Galveston, TX 77555, USA; 13 University of Texas MD Anderson Cancer Center, Houston, TX 77030, USA; 14 Department of Ophthalmology, The University of Iowa Hospitals and Clinics, Iowa City, IA 52242, USA; 15 Center for Space Medicine, Baylor College of Medicine, Houston, TX 77030, USA

**Keywords:** National Football League, dynamic visual acuity, visualization engineering, virtual reality, vestibulo-ocular reflex, visual field

## Abstract

The ability to make on-field, split-second decisions is critical for National Football League (NFL) game officials. Multiple principles in visual function are critical for accuracy and precision of these play calls, including foveation time and unobstructed line of sight, static visual acuity, dynamic visual acuity, vestibulo-ocular reflex, and sufficient visual field. Prior research has shown that a standardized curriculum in these neuro-ophthalmic principles have demonstrated validity and self-rated improvements in understanding, confidence, and likelihood of future utilization by NFL game officials to maximize visual performance during officiating. Virtual reality technology may also be able to help optimize understandings of specific neuro-ophthalmic principles and simulate real-life gameplay. Personal communication between authors and NFL officials and leadership have indicated that there is high interest in 3D virtual on-field training for NFL officiating. In this manuscript, we review the current and past research in this space regarding a neuro-ophthalmic curriculum for NFL officials. We then provide an overview our current visualization engineering process in taking real-life NFL gameplay 2D data and creating 3D environments for virtual reality gameplay training for football officials to practice plays that highlight neuro-ophthalmic principles. We then review in-depth the physiology behind these principles and discuss strategies to implement these principles into virtual reality for football officiating.

## 1. Introduction

In professional American football, split-second decisions in real-time are an integral facet of successful gameplay and game officiating. Various factors enable National Football League (NFL) officials to make these critical gametime decisions that rely on various factors, most notably their vision, visual processing, and positioning to view the play. NFL game officials must make conscious decisions to move—or not move—their bodies on any individual play, and that decision can impact the accuracy and precision of play-calling. Failure to optimize on-field positioning may result in inaccurate calls because of an insufficient visual field, obstructed line of sight, delayed visual processing, or a degradation of foveation time during dynamic visual acuity. In recent years, video assistant systems have been used to help supplement game official calls, but limits on spatial resolution and an inherent time delay indicate that they cannot be a replacement for in-game official play calls [1]. Instead, game officials must make informed decisions on the need for head or body movement in real time, and we believe that understanding neuro-ophthalmic principles and practice can help to improve the ability to identify and process relevant visual cues. Previous studies on the importance of neuro-ophthalmic principles and visual skills on game officials’ performances have emphasized that these principles may have a significant impact on the accuracy of play calls [2,3,4]. By understanding and implementing these neuro-ophthalmic principles in decision making, officials may employ these fundamentals during on-field play-calling [5]. Additionally, the advent of virtual reality technology may be able to further optimize neuro-ophthalmic training and gameplay simulation for football officials.

**Figure 1:**
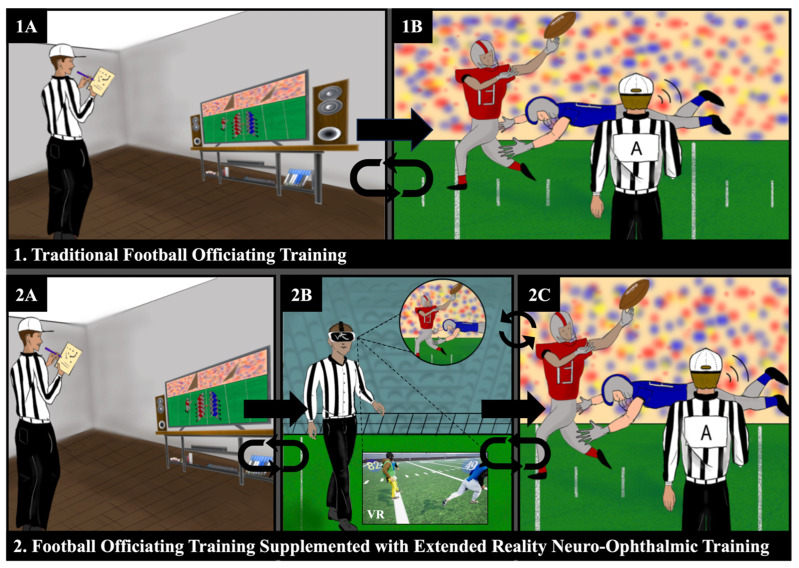
Framework for potential football officiating training with extended reality. Traditional officiating (**1A**,**1B**) includes watching gameplay analysis followed by on-field experience. Officiating training with extended reality (**2A**,**2B**,**2C**) may include gameplay analysis followed by on-field, actively engaged, extended reality experience to simulate difficult plays that occurred in real life. This can be cycled with gameplay until officials are comfortable with on-field officiating at any level, particularly when starting to officiate at a higher stakes level.

In a previous study, a pilot neuro-ophthalmic curriculum was presented to senior NFL game officials through both virtual learning and in-person content. The pilot program demonstrated initial success by establishing that understanding the visual science behind NFL official training improves self-assessed confidence and knowledge that might improve accuracy and precision for in-game play calling in the future [5]. During the in-person NFL neuro-ophthalmology symposium, pre-course and post-course surveys were taken from 21 participants regarding their level of understanding of oculomotor terms and the likelihood of using oculomotor terms. In addition, survey questions on the relevance of these neuro-ophthalmic principles to teaching NFL officials and interest in additional content for future teachings were also recorded. The pre-course rating for the level of understanding of oculomotor terms was 3.4 (out of 10), and the post-course rating was 8.9. For the likelihood of using ocular motor terms covered in the lecture, the pre-course rating was 3.2, and the post-course rating was 8.8. Participants in the survey also self-rated the relevance of the material to their NFL official coaching and teaching a 9.2 and their interest in additional content was rated a 9.4, indicating that integrating neuro-ophthalmic principles in NFL game official training may be beneficial in improving accuracy and precision for play calls on the field [5]. More recently, authors JO, NVC, and AGL traveled to Dallas, Texas to attend a “face-to-face” meeting at the NFL Official Training Camp in Irving, Texas held on 8 and 9 September 2023 [6]. Personal communication between authors and NFL officiating personnel prior to and during this meeting indicated that there was a high interest in virtual reality simulation for NFL officiating training. As such, efforts in visualization engineering of a virtual world environment specifically for NFL officials have been initiated. In this manuscript, we first provide an overview of the neuro-ophthalmic principles (dynamic visual acuity, vestibulo-ocular reflex, visual field) that have been introduced in the NFL neuro-ophthalmic curriculum. We then describe current developments and research in football officiating simulations, virtual and real neuro-ophthalmic training, and the applications and limitations of these neuro-ophthalmic principles in the virtual reality space (Figure 1). We then provide an in-depth overview of the physiology of the neuro-ophthalmic fundamentals and integrate these fundamentals into this 3D virtual on-field application for NFL officiating.

## 2. Introduction to Neuro-Ophthalmic and Football Officiating Terminology

In this section, we briefly introduce the neuro-ophthalmic principles and several NFL officiating terminologies to provide context to the 3D virtual reality environment development. In a subsequent section, we review more in-depth the underlying physiology behind these principles. The neuro-ophthalmic principles discussed in the curriculum include static visual acuity (SVA), dynamic visual acuity (DVA), minimum position error (MPE), saccades, smooth pursuit, visual field, and the vestibulo-ocular reflex (VOR). SVA is defined as the ocular system’s ability to differentiate fine distinctions in the environment between stationary objects [7,8]. DVA is the ability to differentiate these details in the environment while there is relative movement of the observer (i.e., the NFL game official) and their target [9,10,11]. Understanding this distinction between SVA and DVA is critical for NFL officials to make intentional decisions regarding body movement during NFL gameplay. DVA can be assessed with three methods: (1) The participant’s head is fixed and the eyes are tracking a moving target; (2) The target is fixed, and the head and eyes move; (3) The participant’s both head and eyes move to track a moving target [12]. The participant’s head fixed and the eyes tracking is an example of smooth ocular pursuit task with possible catch-up saccades [13]. When the head is moving with a stationary fixation object, this is primarily a VOR task [13]. When the head and eyes are tracking, this employs smooth head movement, smooth eye movement, and catch saccades. It is also critical to note that this latter task requires the normal VOR to be suppressed [13]. An example of VOR suppression in athletics is during the event of batting in baseball for which the head requires VOR suppression [13]. The intricacies of DVA and its relation to VOR and VOR suppression are complex. For example, following a moving football player on the field as an NFL official requires smooth head and eye pursuit, as well as VOR suppression, but VOR must still be at a functional level to be able to mitigate high-frequency head movements when the feet hit the ground while running [13]. As DVA is a complex visual function with various methods of measurement, Murray et al. analyzed these three protocols to measure DVA on a specific DVA test by analyzing its reliability [14]. The authors found that the specific DVA test to test all three protocols resulted in high reliability [14]. Fast movements, such as running, while tracking a play cause a degradation of foveation time and visual acuity (DVA), which can impact the accuracy and precision of call making while on the field. For example, an official’s visual acuity (VA) is degraded most if they must run to get into position to track a stationary target. VA is degraded the least if the official is stationary while the target is also stationary. Similarly, DVA can degrade VA when both the official and the target are in motion. This is because the more movement there is between the subject and the target, the more of a corrective vestibulo-ocular response is required to stabilize the image on the fovea and maintain foveation time [15]. To maximize VA, NFL officials should aim to minimize the need for this corrective stabilization by remaining stationary when possible or minimizing necessary movement by pivoting instead of running.

In addition to DVA, minimum position error (MPE), saccadic prioritization to targets, smooth pursuit, the vestibulo-ocular reflex (VOR), and the visual field may have a notable influence on decision making for NFL officials’ movement on the field. Position error is the difference between the position of the eye and the position of the target. MPE is a metric that corresponds to the alignment of the gaze with a moving object, such as gaze on a football being thrown in the air [15]. Smooth pursuit and saccades both relate to extraocular muscle movement to target and, subsequently, to track objects of fixation. Smooth pursuit (e.g., following a receiver downfield) involves slower target tracking in order to keep the target on the fovea as it moves. Saccades are more rapid movements that quickly move the fovea from one target (e.g., the football) to a new target (e.g., quarterback pass to a wide receiver) [16]. Both saccade and pursuit movements are necessary to track objects during NFL officiating. Sometimes, catch-up saccades replace smooth pursuit at high target speeds to reduce lag while tracking, but this can lead to errors as a result of motion blur [15,17]. Saccadic suppression is a complex central nervous system mechanism to suppress sensory information, which allows for maintenance of stable perception. However, suppression of sensory information during saccadic suppression has an impact on visual acuity and may impact play calling and decision making [18]. NFL officials are trained to remain stationary or minimize unnecessary motion (i.e., “move with purpose”) when possible. Remaining stationary (assuming unobstructed line of sight and sufficient visual field) allows for MPE when foveating and then tracking a moving object during play development (e.g., the football). By moving in such a way to maximize eye movement control, for example, minimizing position error or reducing motion blur from saccades, the NFL official can reduce the chance of degradation of DVA [15]. 

Saccades are also necessary during football plays when predicting future play (e.g., saccade prioritization to developing “mesh points”). A mesh point during game action is the location in which the critical part of the specific play converges (e.g., at the point of a completed pass reception). An individual game official may be tasked with determining a completion or incompletion of the catch—an in or out of bounds reception—and the presence or absence of an interference penalty during the pass play. Understanding and predicting these mesh points through saccadic prediction and prioritization is crucial to making accurate calls. The involved game official must have appropriate saccadic prioritization to the mesh point; an unobstructed line of sight on the play; a sufficient visual field and minimized effects of DVA for an accurate call on the field.

The VOR is compensatory eye movements to stabilize the eye in response to rapid head movements. This occurs through input to the semicircular canals leading the eyes to move in the opposite direction that the head is moving, which preserves the foveal stability of the image on the retina [19]. Movements of the head, such as turning or running, both engage the VOR, leading this reflex to be activated often during NFL officiating. The VOR may also play a role in DVA, particularly in cases in which both the official and the target are in motion and the VOR is needed to stabilize the official’s image of the target as they move. As there are multiple ways to assess DVA, VOR may be analyzed when the target is fixed but the head and eyes are both moving [19]. 

## 3. 3D Environment Virtual Reality Research for Football Officiating

The emergence of virtual reality technology has revolutionized many fields, including sports and medicine [20,21,22,23,24,25,26,27]. Research in virtual reality has found that decision making in sub-elite soccer officials between on-field and virtual reality were indistinguishable, suggesting virtual reality offers a representative training environment [28]. Questionnaire-based research has also indicated a positive response towards a virtual reality training environment for soccer officials [29], paralleling aspects of the personal communication from the NFL officials’ meeting regarding virtual reality training for NFL officiating. The other literature regarding this topic also highlight virtual reality’s ability to simulate various constraints, including crowd noise and other in-game factors, allowing novice officials to have more representative training to strengthen in-game decision making [30,31]. This constraints-based perspective in employing virtual reality technology for use in officiating training may be a solution to overcome certain barriers that novice officials may traditionally face [30]. In baseball, training in the virtual batting environment has led to improvement in real-time, on-field performance, further supporting the positive impact of the virtual reality environment for training [32]. In this section, we discuss our work in building a 3D virtual reality environment based on real-life NFL play data for simulation and neuro-ophthalmic training for football officiating. We also provide a discussion on the integration of this technology with physiology.

We first demonstrate our approach on how we can generate a three-dimensional (3D) explorable environment from limited information about player location and motion without any pre-existing view. Future work can then iteratively refine that 3D scene with information from 2D views.

If we believe that a full game simulation requires P_Team and B_G, information about each player in the field of play and the ball, respectively, then 3D reconstruction can be achieved by synthesizing any view *V* from these sets of information using some reconstruction function *F*:

$$\;V=F(\sum_{i=1}^{n}P_{HomeTeam(i)},\sum_{i=1}^{n}P_{AwayTeam(i)},P_{Ball})$$

However, collecting all motion, pose, and location data for every player with high precision and frequency is itself a non-trivial task. To that end, we constrain ourselves to using only the information available in the dataset and seeing how closely the resulting simulation can replicate real gameplay. As such, we introduce a term called *G_mechanics* that encodes team and event-related information so that we can adequately synthesize a scene without having access to higher precision and frequency of player pose, motion, and location.

$$\tilde{V}=F(G\_mechanics(\sum_{i=1}^{n}{LP}_{HomeTeam(i)},\sum_{i=1}^{n}{LP}_{AwayTeam(i)}),{LP}_{Ball})$$

For now, *G_mechanics* simply uses the distance covered between consecutive frames to predict the player’s pose and animation. But future work should use more play-aware conditions, such as penalty events and neighboring player animation, to construct more precise animations and poses.

Here, we collect the ${LP}_{Team(i)}$ component across three publicly available, real-life sports datasets that showcase individual player movement during a real game. Although this paper is focused on football, we utilized large, real-life 2D datasets from various professional sports to build ${LP}_{Team(i)}$. Later, we discuss preprocessing the datasets to ensure that the formats are consistent amongst different sports (e.g., halves and quarters in different sports). This approach allows for including multiple datasets that do not necessarily need to be in one sport. These datasets include the following:

1. NFL Play Data: NFL Big Data Bowl 2022 [33];
2. NBA Play Data: NBA Player Movements [34];
3. Soccer Data: SoccerTrack [35].

${LP}_{Team(i)}$ consists of the following information:

1. Player location information primarily in the form of x and y coordinates with respect to the field of play. As such, this must be scaled for representation inside our 3D environment;
2. Player jersey number;
3. Player team;
4. Only in the NFL data set additional information such as player speed, acceleration and distance covered was included;
5. None of these datasets contained any explicit pose data;
6. The soccer dataset had accompanying 2D videos and the NBA and NFL video data can be accessed through official channels if required.

We then preprocessed the dataset to ensure that the format is suitable and consistent across the different plays of a single game (first and second halves), as well as different plays of different sports (first half of soccer and third and fourth quarters of NBA). All the preprocessing code is available upon reasonable request. The extracted information was stored in csv files for each play for each team and player across different time indices. All csv files are available upon reasonable request. For 3D visualization, we read and employed the csv files in Unreal Engine 5.3. We created an extension of “Actor” called “FileFormation” that extracts all relevant information to construct LP_Team and LP_ball by taking into account the size of the stadium that the players are shown in. An instance of that object is placed inside the level with the topLeft and bottomRight being adjusted to encompass the field. 

**Figure 2:**
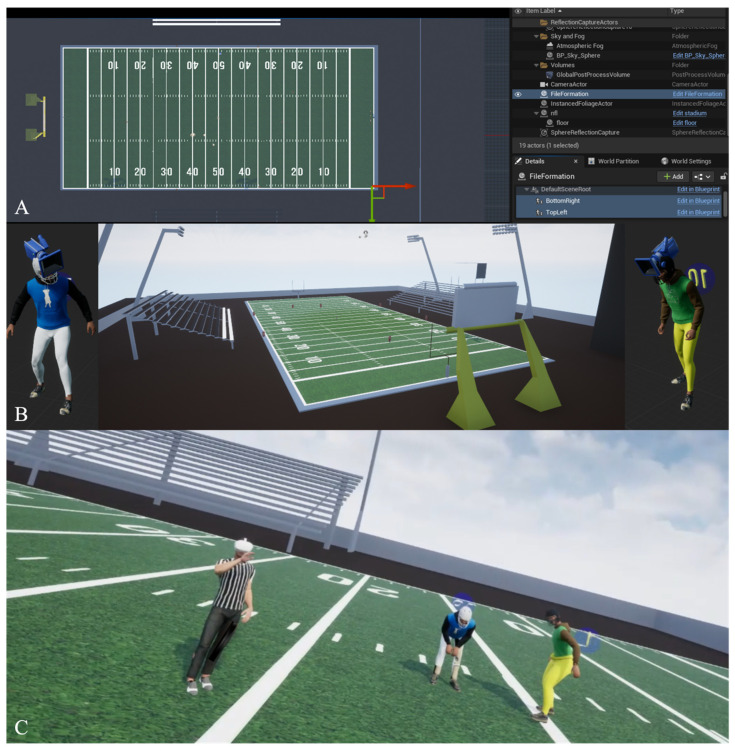
Stepwise visualization engineering framework for the 3D environment for football officiating training and simulation using real-life NFL play 2D data. Panel (**A**) demonstrates preprocessed data from real-life 2D NFL gameplay data that map out every individual on the field at specific time indices. Panel (**B**) showcases the 3D generation of “Actors” and the ball on the field through Unreal Engine that can play out real-life NFL play data. Panel (**C**) showcases the point of view on the field that can interact with real-life NFL play data and can be utilized with wearable virtual reality for NFL officiating VR simulation and training.

When a specific game is selected, we instantiate each player at the initial location in the field of play according to their team and jersey. The initial player orientation and animation is selected based on subsequent frames. We leverage the MetaHuman plugin by Unreal Engine to generate a random body and face furnished with team-specific jerseys. The animations are modified sequences of third-person animation available as default. Finally, different referee players are also created at predefined locations where a subject can embody them or virtual cameras at different relative or static locations across the field to explore the 3D gameplay (Figure 2). Individuals can switch between different cameras using the following keys: Referees (Numpad 1,2,4,7,8,9,6); Perspective Camera (Numpad 3); Top-down view with respect to the ball (Numpad 5); Random player from either team (Numpad 0). All the UnrealEngine code is available upon reasonable request.

## 4. Physiology of Neuro-Ophthalmic Principles for 3D Virtual On-Field Training

In this section, we discuss the physiology of neuro-ophthalmic principles and its potential to be integrated in virtual reality training for football officiating. We also discuss current research that has been conducted in DVA assessment and augmentation in virtual reality.

The visual pathways that allow NFL officials to make split-second decisions in real time rely on a system more complex than the traditional visual acuity tests. Assessing visual acuity through static optotypes, which involves viewing letters or numbers of varying sizes with perfect contrast for an extended time, certainly produces a specific visual acuity metric. While some penalties in football occur while the official and players are static (e.g., illegal formation and illegal shift), many penalties occur after the ball is snapped and while the play is in motion. For referees making those calls, this type of vision relies on DVA, representing the ability to visualize objects when there is relative movement between the subject and the object [15,36].

DVA plays a crucial role in many ball sports, and research has found a close relationship between DVA and level of expertise [15]. Because DVA is so frequently encountered on the NFL field, having a quantifiable assessment can better aid in understanding officials’ visual acuity and aid in creating programs that increase visual performance. Various tests are utilized to determine DVA, including static optotypes, moving optotypes, and assessments conducted during movements such as head rotation or walking on a treadmill [36,37]. These assessments provide a DVA which can then be compared to static VA in order to provide a more comprehensive evaluation of visual acuity. It is common for VA to decrease between static VA and DVA, but a reduction in more than three lines may indicate an abnormality [38]. While the various tests for DVA do provide more information regarding visual acuity, many studies analyzing DVA are not consistent in their methodology. As such, it is difficult to compare DVA assessments across studies. An emerging field for these tests uses virtual reality to evaluate DVA, which has demonstrated clinical utility when compared to traditional clinical testing [39,40]. A standardized DVA test, through virtual reality or other validated means, can help evaluate the benefits of visual training and create a more robust understanding of the visual impacts of DVA [41].

**Figure 3:**
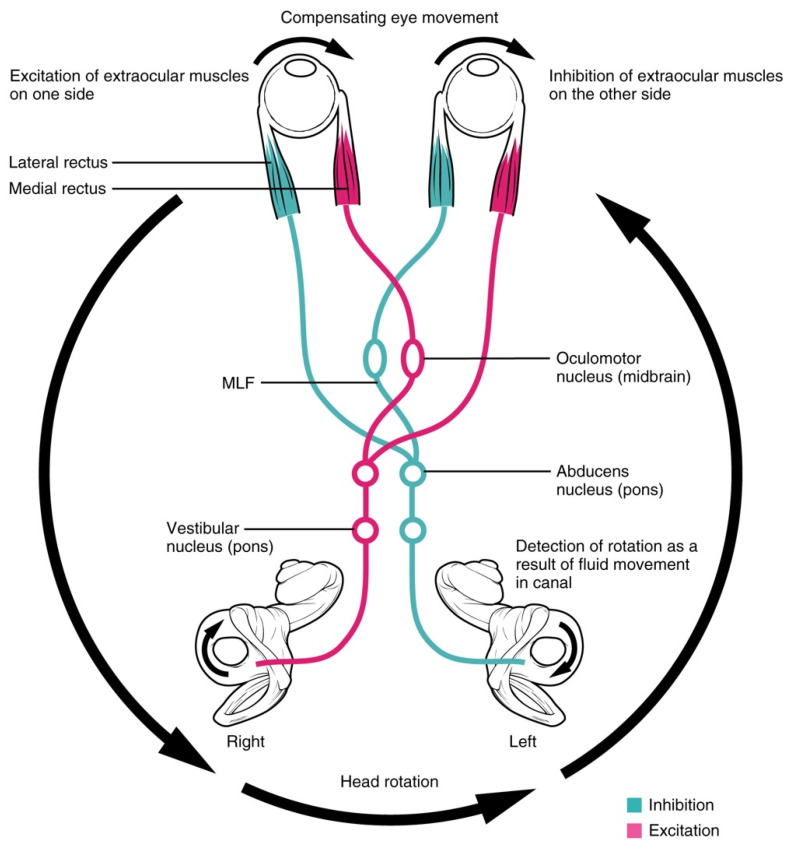
Mechanism of the vestibulo-ocular reflex for horizontal eye movement. Rotation of the head generates a reflexive pathway for compensating eye movement. MLF = Medial longitudinal fasciculus. Reprinted with permission from CFCF in Wikimedia Commons under Creative Commons Attribution 3.0 Unported license (https://creativecommons.org/licenses/by/3.0/legalcode.en; accessed on 1 October 2023.

Furthermore, DVA is closely linked to the VOR, which aids in vision stabilization during head movement. DVA tests that include head movements greater than 2 Hz can activate the VOR [37]. The VOR initiates a compensatory response to head movement that keeps the eyes focused on an object during head movement, making it a crucial part of DVA. During live plays, officials often rotate their head to view the play while keeping their gaze fixed at a specific point. This head movement pattern requires the use of the VOR to stabilize the image on the fovea. The VOR consists of three major components: sensors in the periphery, central processing, and the muscle output [42]. The semicircular canals are the peripheral sensors responsible for detecting head rotation and angular acceleration. These sensory organs consist of six canals that connect with the six extraocular muscles (the muscle output) through the abducens nucleus, oculomotor nucleus, and medial longitudinal fasciculus (central processing) [43]. The excitatory signal from the six canals then converts the head motion into a neural signal that initiates eye movements to stabilize a moving image on the fovea. This description describes the VOR mechanism for horizontal responses. Figure 3 displays the connections in the VOR and how the compensatory horizontal eye movements are initiated.

The VOR is an integral part of sports performance, and its adaptation varies across different sports. Research comparing athletes in combat sports against those in ball sports found that athletes in ball sports have a superior VOR [43]. While there is less research specifically assessing the VOR of NFL officials, the requirement for well-adapted visual processing is apparent, as they must continuously track players, balls, and field positions while in motion.

Although the VOR system is excellent at visual stabilization, there is a 5–8 ms delay between head movement and eye rotation response in a healthy individual [44]. This lag produces a brief delay in what the official sees and their visual processing. Disruptions that further increase this delay can result in nausea, head tilt, and blurred vision during motion [45]. Because of these disturbances, adaptations of the VOR have been extensively studied, mainly in relation to vestibular disorders. Mahfuz et al. discusses basic principles of VOR adaptation training and the creation of a vestibulo-visual mismatch stimulus through the use of lenses, prisms, moving visual displays, or a moving visual laser target. The basic principle for their lens is to increase the visual movement for a given head rotation so that the VOR eye movement is not able to stabilize the image on the fovea [46]. The mismatch created from these techniques drives adaptation by producing a concept called “retinal slip” in which the image is not sufficiently stabilized [47]. This retinal slip can also occur during rapid head movements, typically those greater than 2–4 degrees/s, which can decrease DVA [48]. A Cochrane review on vestibular rehabilitation for unilateral peripheral visual dysfunction found that vestibular rehabilitation improved balance and vision. This review was focused on individuals with dysfunction but provides evidence that improvements to the VOR can also improve vision [49].

Another decision-making factor that is critical in officiating is the concept of anticipation [50,51]. The concept of anticipation allows for officials to employ their knowledge structures to become efficient in their visual search to anticipate where to locate critical details in an upcoming event [52,53]. Advanced anticipation and visual search skills have been established in the literature as a critical quality for football officials at the elite level [50,54]. Mastery over this concept allows officials to optimize their view at the most critical location prior to an event (e.g., a possible foul). Bieman et al. employed eye-tracking techniques between elite and sub-elite officials that showed differences in eye gaze directions to produce towards earlier anticipation ocular movements [50]. Schrödter et al. also found superior anticipation in elite referees compared to non-elite referees in basketball [53]. The concept of anticipation in officiating is very closely linked to the neuro-ophthalmic principles discussed above. The results of these studies indicate that official expertise may play a role in the implementation of these neuro-ophthalmic principles. Additionally, the neuro-ophthalmic principles may be integrated with longstanding knowledge structures to amplify decision-making skills on the field. In a similar vein of research, Baptista et al. found that after adjusting for experience and demographic factors in soccer officials, stereoacuity and visual memory were the factors that were associated with superior on-field performance [55]. It was also found that roughly a quarter of elite soccer officials in a study had vision that could be capable of improved visual acuity while officiating. These factors may also impact the integration of neuro-ophthalmic principles on the field [56]. These points are areas that may be further explored in research and may help to further understand the integration of neuro-ophthalmic principles with complex knowledge structures from years of officiating.

**Figure 4:**
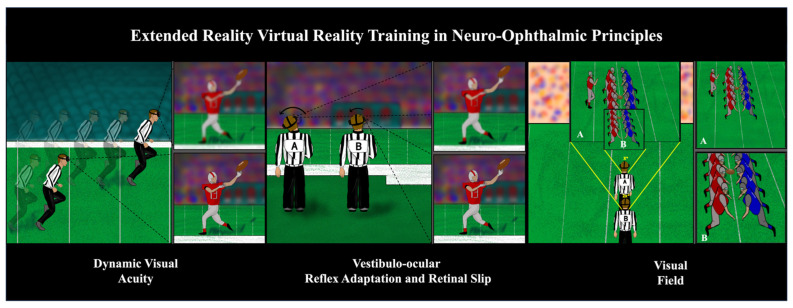
Implementation of virtual reality training in neuro-ophthalmic principles to showcase changes in dynamic visual acuity based on running speed and vestibulo-ocular reflex adaptation, as well as retinal slip based on acceleration of head tilt and visual field changes based on positioning.

With the implementation of “Move With Purpose”, a teaching philosophy for NFL officials to move on the field with clear intentions, NFL game officials are being trained to reduce body movement during play calls (Personal Communication, Walt Anderson, Senior Vice President of NFL Officiating Training and Development). However, officials are still in situations where movement is required to make crucial calls. In these moments, officials must depend on their VOR and DVA to maintain focus on the play, as well as their positioning, to optimize their visual field. Unlike the high-quality, expansive, and static field of view most spectators enjoy when they ask the officials, “What game were you watching?”, officials may have moments of reduced visual acuity that occurs in motion as well as a VOR delay. This complex visual processing results in lower-quality images than those obtained through a slow-motion instant replay. Innovative virtual reality technology can help officials to further understand these concepts on the field and may provide tools to further build knowledge bases and inferences for in-game decision making [57]. Additionally, current virtual reality technology contains eye-tracking technology that can be useful for officials in understanding their gazes, and there is ongoing research to understand how virtual reality can augment DVA [58,59]. Superior DVA has been found to be due to superior tracking abilities in baseball players; thus, eye-tracking technology may provide unique insights into DVA ability for NFL officiating [60]. Eye tracking may also provide further insights into an officials ability to identify mesh points by analyzing gaze [4,28]. Figure 4 demonstrates how virtual reality technology can simulate changes in (1) DVA based on running speed; (2) VOR adaptation and retinal slip based on acceleration of head-tilt; and (3) visual field changes based on positioning. By implementing the “Move With Purpose” approach and neuro-ophthalmic virtual reality training, including DVA, VOR, and visual field training, NFL officials can cultivate skills that enhance their visual processing capabilities and minimize visual disruptions.

## 5. Conclusions

Prior work has shown the high level of interest in neuro-ophthalmic curriculum regarding the topics discussed in this manuscript for NFL officiating [5]. A firm understanding by NFL game officials of the visual function and performance factors during officiating is important to further understand how to optimize on-field decision making. Based on personal communications at the 2023 NFL Officials’ meeting in Dallas, Texas, there is high interest in 3D virtual on-field training that can implement these neuro-ophthalmic fundamentals. We plan to continue developing and implementing technology for teaching and assessing in 3D virtual reality environments with real NFL play data in our future curriculum for NFL game officials. Quantifiable metrics, including decision-making call similarities between on-field and virtual reality environments, must be analyzed to determine the efficacy of this technology. Full future integration of virtual reality training using these neuro-ophthalmic principles will hopefully improve the precision and accuracy of NFL play calling. Modifications and use of current off-the-shelf virtual reality technology is an exciting opportunity to create and optimize gameplay simulation and training for football officiating at the highest level, which might be generalizable and scalable across the spectrum of football and other sports.

## Data Availability

Data are contained within the article.

## References

[1] Mather G. (2020). A Step to VAR: The Vision Science of Offside Calls by Video Assistant Referees. Perception.

[2] Ghasemi A., Momeni M., Jafarzadehpur E., Rezaee M., Taheri H. (2011). Visual skills involved in decision making by expert referees. Percept. Mot. Ski..

[3] Aginsky K.D., Noakes T.D. (2008). Why it is difficult to detect an illegally bowled cricket delivery with either the naked eye or usual two-dimensional video analysis. Br. J. Sports Med..

[4] Ziv G., Lidor R., Zach S., Brams S., Helsen W.F. (2020). Gaze Behavior of Referees in Sport—A Review. Front. Sports Act. Living.

[5] Vogt A.Z., Woodland M.B., Carter M.J., Lee A.G. (2023). Curriculum in Neuro-Ophthalmic Principles for National Football League Game Officials: Comparison of Pretraining and Posttraining Ratings of Knowledge. J. Neuro-Ophthalmol..

[6] 2023 NFL Game Officials’ Meeting, Irving, TX, USA, 8–9 September 2023. https://operations.nfl.com/officiating/nfl-officials-preparing-for-success/.

[7] Burg A. (1966). Visual acuity as measured by dynamic and static tests: A comparative evaluation. J. Appl. Psychol..

[8] Ong J., Tavakkoli A., Strangman G., Zaman N., Kamran S.A., Zhang Q., Ivkovic V., Lee A.G. (2022). Neuro-ophthalmic imaging and visual assessment technology for spaceflight associated neuro-ocular syndrome (SANS). Surv. Ophthalmol..

[9] Jorge J., Fernandes P. (2019). Static and dynamic visual acuity and refractive errors in elite football players. Clin. Exp. Optom..

[10] Waisberg E., Ong J., Paladugu P., Kamran S.A., Zaman N., Lee A.G., Tavakkoli A. (2023). Dynamic visual acuity as a biometric for astronaut performance and safety. Life Sci. Space Res..

[11] Zaman N., Waisberg E., Ong J., Paladugu P., Kamran S.A., Lee A.G., Tavakkoli A. (2023). Poster Session: Comparison of Dynamic Visual Acuity Assessments in Head-Mounted Technology and Traditional Laptop-based Method. J. Vis..

[12] Riska K.M., Hall C.D. (2016). Reliability and Normative Data for the Dynamic Visual Acuity Test for Vestibular Screening. Otol. Neurotol..

[13] Toole A.J., Fogt N. (2021). Review: Head and Eye Movements and Gaze Tracking in Baseball Batting. Optom. Vision Sci..

[14] Murray N., Hunfalvay M., Roberts C.-M., Lange B. (2017). Reliability and normative data of computerized dynamic visual acuity tests. Vision Dev. Rehabil..

[15] Palidis D.J., Wyder-Hodge P.A., Fooken J., Spering M. (2017). Distinct eye movement patterns enhance dynamic visual acuity. PLoS ONE.

[16] Leigh R.J., Kennard C. (2004). Using saccades as a research tool in the clinical neurosciences. Brain.

[17] Orban de Xivry J.J., Lefevre P. (2007). Saccades and pursuit: Two outcomes of a single sensorimotor process. J. Physiol..

[18] Crevecoeur F., Kording K.P. (2017). Saccadic suppression as a perceptual consequence of efficient sensorimotor estimation. eLife.

[19] Bronstein A.M., Patel M., Arshad Q. (2014). A brief review of the clinical anatomy of the vestibular-ocular connections—How much do we know?. Eye.

[20] Zaman N., Ong J., Waisberg E., Masalkhi M., Lee A.G., Tavakkoli A., Zuckerbrod S. (2023). Advanced Visualization Engineering for Vision Disorders: A Clinically Focused Guide to Current Technology and Future Applications. Ann. Biomed. Eng..

[21] Ong J., Tavakkoli A., Zaman N., Kamran S.A., Waisberg E., Gautam N., Lee A.G. (2022). Terrestrial health applications of visual assessment technology and machine learning in spaceflight associated neuro-ocular syndrome. NPJ Microgravity.

[22] Sarker P., Zaman N., Ong J., Paladugu P., Aldred M., Waisberg E., Lee A.G., Tavakkoli A. (2023). Test-Retest Reliability of Virtual Reality Devices in Quantifying for Relative Afferent Pupillary Defect. Transl. Vis. Sci. Technol..

[23] Nolin P., Stipanicic A., Henry M., Joyal C.C., Allain P. (2012). Virtual reality as a screening tool for sports concussion in adolescents. Brain Inj..

[24] Faure C., Limballe A., Bideau B., Kulpa R. (2020). Virtual reality to assess and train team ball sports performance: A scoping review. J. Sports Sci..

[25] Ong J., Hariprasad S.M., Chhablani J. (2022). Into the RetinaVerse: A New Frontier of Retina in the Metaverse. Ophthalmic Surg. Lasers Imaging Retin..

[26] Brent Woodland M., Ong J., Zaman N., Hirzallah M., Waisberg E., Masalkhi M., Kamran S.A., Lee A.G., Tavakkoli A. (2024). Applications of extended reality in spaceflight for human health and performance. Acta Astronaut..

[27] Ong J., Zaman N., Waisberg E., Kamran S.A., Lee A.G., Tavakkoli A. (2022). Head-mounted digital metamorphopsia suppression as a countermeasure for macular-related visual distortions for prolonged spaceflight missions and terrestrial health. Wearable Technol..

[28] van Biemen T., Müller D., Mann D.L. (2023). Virtual reality as a representative training environment for football referees. Hum. Mov. Sci..

[29] Gulec U., Yilmaz M., Isler V., O’Connor R.V., Clarke P.M. (2019). A 3D virtual environment for training soccer referees. Comput. Stand. Interfaces.

[30] Kittel A., Lindsay R., Larkin P., Spittle M. (2022). The application of 360°VR for training sports officials: A constraints-led approach. Manag. Sport Leis..

[31] Kittel A., Larkin P., Elsworthy N., Spittle M. (2019). Using 360° virtual reality as a decision-making assessment tool in sport. J. Sci. Med. Sport.

[32] Gray R. (2017). Transfer of Training from Virtual to Real Baseball Batting. Front. Psychol..

[33] NFL (2022). NFL Big Data Bowl 2022. Kaggle. https://www.kaggle.com/competitions/nfl-big-data-bowl-2022/data.

[34] Linou K., Linou D., de Boer M. (2016). NBA Play Data. Github. https://github.com/linouk23/NBA-Player-Movements/tree/master.

[35] Scott A., Uchida I., Onishi M., Kameda Y., Fukui K., Fujii K. SoccerTrack: A Dataset and Tracking Algorithm for Soccer with Fish-eye and Drone Videos. Proceedings of the 2022 IEEE/CVF Conference on Computer Vision and Pattern Recognition Workshops (CVPRW).

[36] Wu T.Y., Wang Y.X., Li X.M. (2021). Applications of dynamic visual acuity test in clinical ophthalmology. Int. J. Ophthalmol..

[37] Chen G., Zhang J., Qiao Q., Zhou L., Li Y., Yang J., Wu J., Huangfu H. (2022). Advances in dynamic visual acuity test research. Front. Neurol..

[38] Erdinest N., London N. (2022). Dynamic visual acuity and methods of measurement. J. Optom..

[39] Holford K.C., Jagodinsky A.E., Saripalle R., McAllister P. (2022). Leveraging virtual reality for vestibular testing: Clinical outcomes from tests of dynamic visual acuity. J. Vestib. Res..

[40] Waisberg E., Ong J., Zaman N., Kamran S.A., Lee A.G., Tavakkoli A. (2022). Head-Mounted Dynamic Visual Acuity for G-Transition Effects During Interplanetary Spaceflight: Technology Development and Results from an Early Validation Study. Aerosp. Med. Hum. Perform..

[41] Zimmerman A.B., Lust K.L., Bullimore M.A. (2011). Visual acuity and contrast sensitivity testing for sports vision. Eye Contact Lens.

[42] Robinson D.A. (2022). Basic framework of the vestibulo-ocular reflex. Prog. Brain Res..

[43] Kizilay F., Cengiz D.U. (2023). A comparison of functional vestibulo-ocular reflex and proprioception in athletes of combat sports and ball sports. Heliyon.

[44] Figtree W.V.C., Schubert M.C., Rinaudo C.N., Migliaccio A.A. (2020). The instantaneous training demand drives vestibulo-ocular reflex adaptation. Exp. Brain Res..

[45] Simakurthy S., Tripathy K. (2023). Oculovestibular Reflex. StatPearls.

[46] Muntaseer Mahfuz M., Schubert M.C., Figtree W.V.C., Todd C.J., Migliaccio A.A. (2018). Human Vestibulo-Ocular Reflex Adaptation Training: Time Beats Quantity. J. Assoc. Res. Otolaryngol..

[47] Todd C.J., Schubert M.C., Figtree W.V.C., Migliaccio A.A. (2019). Incremental Vestibulo-ocular Reflex Adaptation Training Dynamically Tailored for Each Individual. J. Neurol. Phys. Ther..

[48] Morimoto H., Asai Y., Johnson E.G., Lohman E.B., Khoo K., Mizutani Y., Mizutani T. (2011). Effect of oculo-motor and gaze stability exercises on postural stability and dynamic visual acuity in healthy young adults. Gait Posture.

[49] McDonnell M.N., Hillier S.L. (2015). Vestibular rehabilitation for unilateral peripheral vestibular dysfunction. Cochrane Database Syst. Rev..

[50] Van Biemen T., van Zanten T.F., Savelsbergh G.J.P., Mann D.L. (2022). “What needs to be seen”: An exploration into the visual anticipation behaviour of different skill-level football referees while observing long passes on-field. Hum. Mov. Sci..

[51] Hodges N.J., Wyder-Hodge P.A., Hetherington S.M., Baker J., Besler Z.B., Spering M. (2021). Topical Review: Perceptual-cognitive Skills, Methods, and Skill-based Comparisons in Interceptive Sports. Optom. Vis. Sci..

[52] Klatt S., Noël B., Nicklas A., Schul K., Seifriz F., Schwarting A., Fasold F. (2021). Gaze Behavior and Positioning of Referee Teams during Three-Point Shots in Basketball. Appl. Sci..

[53] Schrödter R., Schwarting A., Fasold F., Schul K., Klatt S. (2023). The Relevance of General Spatial Anticipation Skills for Basketball Referees. Appl. Sci..

[54] Slack L.A., Maynard I.W., Butt J., Olusoga P. (2013). Factors Underpinning Football Officiating Excellence: Perceptions of English Premier League Referees. J. Appl. Sport Psychol..

[55] Baptista A.M.G., Serra P.M., Faisal M., Barrett B.T. (2021). Association between Clinical Vision Measures and Visual Perception and Soccer Referees’ On-field Performance. Optom. Vis. Sci..

[56] Baptista A.M.G., Serra P.M., McAlinden C., Barrett B.T. (2017). Vision in high-level football officials. PLoS ONE.

[57] Cunningham I., Roche L., Mascarenhas D. (2023). Using Mobile 360° Video as a Tool for Enhancing Sport Referee Performance: A Case Study. Case Stud. Sport Exerc. Psychol..

[58] Waisberg E., Ong J., Zaman N., Kamran S.A., Lee A.G., Tavakkoli A. (2022). Stroboscopic Augmented Reality as an Approach to Mitigate Gravitational Transition Effects during Interplanetary Spaceflight. Int. J. Aviat. Aeronaut. Aerosp..

[59] Schuetz I., Fiehler K. (2022). Eye Tracking in Virtual Reality: Vive Pro Eye Spatial Accuracy, Precision, and Calibration Reliability. J. Eye Mov. Res..

[60] Uchida Y., Kudoh D., Higuchi T., Honda M., Kanosue K. (2013). Dynamic Visual Acuity in Baseball Players Is Due to Superior Tracking Abilities. Med. Sci. Sports Exerc..

